# Expectations and Experiences of Participating in a Supervised and Home-Based Physical Exercise Intervention in Patients with Head and Neck Cancer during Chemoradiotherapy: A Qualitative Study

**DOI:** 10.3390/curroncol31020066

**Published:** 2024-02-04

**Authors:** Annemieke Kok, Ellen Passchier, Anne M. May, Harriët Jager-Wittenaar, Cindy Veenhof, Remco de Bree, Martijn M. Stuiver, Caroline M. Speksnijder

**Affiliations:** 1 Department of Dietetics, University Medical Center Utrecht, Utrecht University, 3584 CX Utrecht, The Netherlands; 2 Department of Head and Neck Oncology and Surgery, The Netherlands Cancer Institute, 1066 CX Amsterdam, The Netherlands; 3 Center for Quality of Life and Division of Psychosocial Research and Epidemiology, The Netherlands Cancer Institute, 1066 CX Amsterdam, The Netherlands; 4 Julius Center for Health Sciences and Primary Care, University Medical Center Utrecht, Utrecht University, 3584 CG Utrecht, The Netherlands; a.m.may@umcutrecht.nl; 5 Department of Oral and Maxillofacial Surgery, University of Groningen, University Medical Center Groningen, 9713 GZ Groningen, The Netherlands; ha.jager@pl.hanze.nl; 6 Research Group Healthy Ageing, Allied Health Care and Nursing, Hanze University of Applied Sciences, 9747 AS Groningen, The Netherlands; 7 Research Unit Experimental Anatomy, Department Physiotherapy and Human Anatomy, Faculty of Physical Education and Physiotherapy, Vrije Universiteit Brussel, 1050 Brussels, Belgium; 8 Department of Rehabilitation, Physiotherapy Science and Sports, UMC Utrecht Brain Center, University Medical Center Utrecht, Utrecht University, 3584 CX Utrecht, The Netherlands; c.veenhof-2@umcutrecht.nl; 9 Research Group Innovation of Human Movement Care, HU University of Applied Sciences, 3584 CS Utrecht, The Netherlands; 10 Department of Head and Neck Surgical Oncology, University Medical Center Utrecht, Utrecht University, 3584 CX Utrecht, The Netherlands; 11 Department of Oral and Maxillofacial Surgery and Special Dental Care, University Medical Center Utrecht, Utrecht University, 3584 CX Utrecht, The Netherlands

**Keywords:** head and neck cancer, exercise, preference, experience, adherence, compliance

## Abstract

(1) Background: Chemoradiotherapy (CRT) for head and neck cancer (HNC) is associated with severe toxicity resulting in fatigue and weight loss, including loss of skeletal muscle mass. Exercise interventions might positively affect physical fitness and quality of life. Sufficient adherence and compliance rates are necessary for optimal effects. This study aimed to gain insight into expectations and experiences and factors influencing adherence, retention and compliance of HNC patients participating in exercise intervention during CRT. (2) Methods: Consecutive participants were invited for semi-structured interviews, conducted pre- and post-intervention. A deductive approach was used to identify themes and factors influencing adherence, retention and compliance. (3) Results: Thematic saturation was reached after interviewing 14 patients pre-intervention. Five themes were identified: planning and time management, treatment toxicity, motivation to exercise, exercise intervention and supervision by a physiotherapist. The intensity of the treatment schedule and treatment toxicity were important barriers. Facilitators mentioned were physical and emotional benefits, social support as well as the simplicity and home-based setting of the intervention. (4) Conclusions: A personalised approach, considering the individual facilitators and barriers of HNC patients, is important to increase adherence, retention and compliance to exercise intervention and to reap the optimal effects of the program.

## 1. Introduction

Chemoradiotherapy (CRT) for head and neck cancer (HNC) is associated with a high risk of severe toxicities like energy loss, decreased masticatory functioning, dysphagia, xerostomia, taste alteration and nausea and vomiting. These side effects, but also the cancer itself, can complicate physical activity and oral nutritional intake, resulting in fatigue, weight loss and loss of skeletal muscle mass [1,2,3]. Loss of muscle mass is associated with a reduced quality of life (QoL), and also a decrease in physical performance and a worse overall prognosis [4,5]. Therefore, interventions aiming at improving physical functioning, body composition, fatigue and QoL are needed. Exercise interventions during cancer treatment have shown to positively affect physical fitness and quality of life and may improve treatment completion rates [6,7,8]. Supervised exercise interventions appear to be the most effective; however, it remains unclear as to what factors are conclusive regarding, among other things setting, dose and motivation [8].

Optimal effects of implementing exercise as part of HNC care can only be achieved when reaching sufficient, adherence, retention and compliance rates. Patients with HNC are generally less physically active, as part of a suboptimal lifestyle, in comparison with other populations with cancer [5,9]. Also, they show a lack of intention to increase exercise levels, probably due to the fact that they perceive their low physical activity level as already being sufficient and experience physical barriers and low self-efficacy [9]. For patients with HNC, achieving sufficient adherence, retention and compliance to exercise interventions during cancer treatment is challenging [10,11,12,13]. Specific determinants to improve feasibility and to establish a tailored approach to increase exercising in this population should be further investigated [14]. Previous qualitative studies focused on physical activity and exercise interventions mainly after HNC treatment [9,14,15]. Moreover, these studies did not cover factors influencing the feasibility of exercise interventions during HNC treatment.

This study is part of a feasibility study in which adherence, retention and compliance of a combined supervised and home-based exercise intervention during CRT was evaluated. Our quantitative analysis showed that feasibility was influenced by the timing, intensity and duration of the exercise, as well as the travelling time and planning difficulties [13]. In this qualitative part of our study, we aimed to gain insight into preferences and expectations of patients with HNC before participating, as well as their experiences and satisfaction with this exercise intervention during CRT. Specifically, the objective was to identify factors influencing adherence, retention and compliance from a patients’ perspective.

## 2. Materials and Methods

### 2.1. Ethics

The study was approved by the Medical Ethical Committee of the University Medical Center Utrecht (17-630). All participants signed informed consent prior to the interview. The Consolidated Criteria for Reporting Qualitative (COREQ) Research checklist was used in the preparation of the manuscript [16]. The study was registered at the Dutch National Trial Register (NTR7305).

### 2.2. Setting, Eligibility and Recruitment

The study was conducted at the University Medical Center Utrecht (UMCU) and The Netherlands Cancer Institute (NKI), The Netherlands. Patients with HNC scheduled for CRT were consecutively recruited, either face-to-face or by phone, for participation in our exercise intervention study. For the quantitative part of the feasibility study [13], 40 patients were included. For the qualitative part of the feasibility study, which is described in this paper, consecutive sampling was used until data saturation was reached.

### 2.3. Exercise Intervention

**Figure 1:**
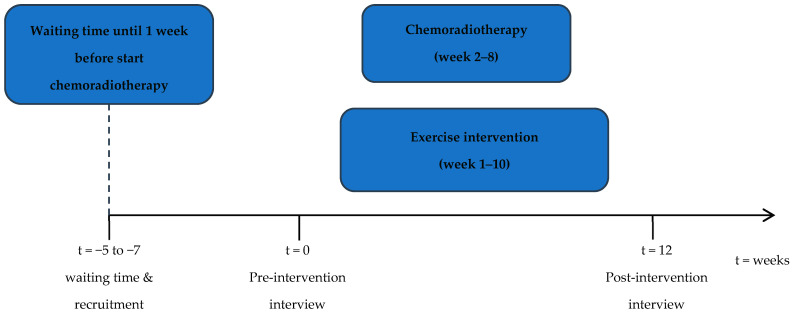
Study flow chart.

The exercise intervention consisted of a 10-week combined endurance and resistance training during CRT treatment offered by a physiotherapist. Nutritional support was offered by a dietitian as part of usual care in both the UMCU and NKI. The start of the exercise intervention was, preferably, before or in the first week of CRT and ended after 10 weeks (Figure 1). The endurance training consisted of 30 min of moderate-intensity physical exercise which included 15 min brisk walking, and another 15 min of exercise of their own choice. For the resistance training, patients were instructed to perform six exercises three times a week, targeting major muscle groups (arms, legs, shoulders, back and core) using body weight and elastic bands for resistance. Patients attended one session per week at the hospital, supervised by a physiotherapist. The remaining training sessions were home-based. Further details about the exercise intervention including adherence, retention and compliance rate, have been described elsewhere [13].

### 2.4. Pre- and Post-Intervention Interviews

Semi-structured interviews were conducted from December 2017 through to June 2018. Patients were recruited for this qualitative study until data saturation was achieved, which was when no new information could be identified from the last two interviews [17,18].

Two pre-defined interview guides were used for the pre- and post-intervention interviews, respectively. These guides were developed by the research team in an open discussion, using results from previous studies [9,15]. At baseline, questions focused on patients’ expectations regarding their adherence, retention and compliance with the exercise intervention during CRT. The post-intervention interviews focused on their actual adherence, retention and compliance. Questions focused on patients’ satisfaction with the intervention (e.g., setting, frequency, intensity, supervision) and on patients’ attitude, preferences, motivation, opportunities and barriers towards exercising during CRT; additionally, suggestions for improvement were explored. Participants were interviewed at a location of their convenience, either at home or at the hospital. Family members were allowed to be present during the interview, but their perspectives were not collected. Each interview lasted between 30 and 45 min and was audio recorded. Field notes were made. The interviews were conducted by EP, who is a nurse specialist and clinical epidemiologist, or by RG, who is a physiotherapist and master’s student in oncology physiotherapy. Both RG and EP were trained by an experienced researcher (GY) in qualitative methods. No prior relationships existed between the researchers and participants. After the interviews, the audio recordings were transcribed verbatim. Patient characteristics comprising sociodemographic and medical data were collected from a baseline study-specific questionnaire and from medical files.

### 2.5. Data Analysis

We performed a thematic analysis to generate codes from the interview transcripts using a deductive approach in alignment with the interview guides [19]. All interview transcripts were read independently by two researchers (EP and AK), followed by open coding of firstly, the pre-intervention and secondly, the post-intervention interviews. After axial coding, specific codes were identified and labelled, and exemplary quotes were selected. Additional codes were generated after reviewing the third and last interview for both the pre- and post-intervention interviews. Codes and categories were established and discussed during meetings with three authors (EP, AK and CMS), subsequently to identify, discuss and clarify overarching themes. To ensure trustworthiness, codes and categories were cross-checked, until no new themes emerged. Any discrepancies in the analysis were discussed until a consensus was reached. In addition, agreements between extracted themes from the pre- and post-intervention interviews were investigated. The computer software NVivo version 12 (QSR International LLC, Burlington, MS, USA) was used for coding.

## 3. Results

### 3.1. Participants

We reached data saturation after interviewing 14 participants pre-intervention. None of the participants in this qualitative part of the study experienced adverse events due to the exercise intervention. Two were lost to follow up resulting in 12 interviews after the exercise intervention. During two interviews the partners of the interviewees were present. Mean age of the participants was 57 years (SD: 8.7 years), and 11 of the 14 interviewed participants were male. Five participants in this qualitative study did not complete the exercise intervention. Participants were asked to rate their satisfaction with the exercise intervention on a scale from 0 to 10; 11 interviewees responded, with an average of 7.6 (range 5–10). All baseline characteristics are shown in Table 1.

### 3.2. Overview of Findings

**Figure 2:**
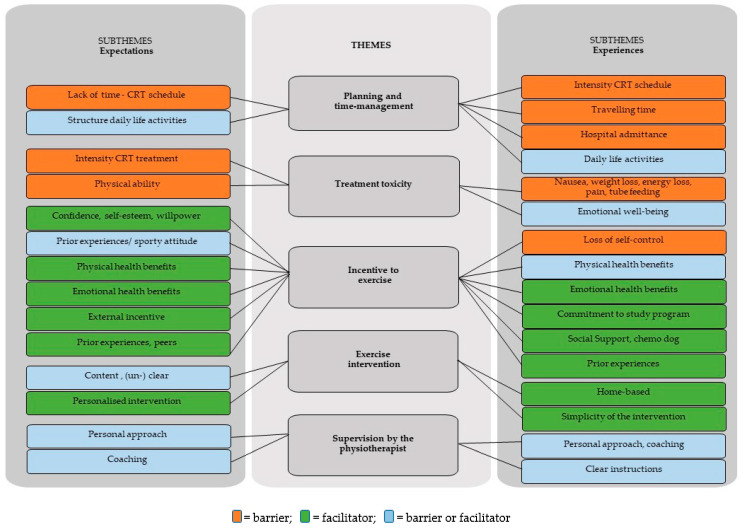
Themes and subthemes extracted from the pre- and post-intervention interviews (representing expectations and experiences, respectively) with head and neck cancer patients participating in exercise intervention during chemoradiotherapy.

From the pre-intervention, referred to as expectations, and post-interventions interviews, referred to as experiences, we extracted five overarching themes: (1) planning and time-management, (2) treatment toxicity, (3) motivation to exercise, (4) exercise intervention and (5) supervision by the physiotherapist. Figure 2 shows the five themes related to both the expectations and experiences of the participants of this study, and the subthemes representing underlying factors. In Table 2, explanatory quotes regarding the (sub)themes are depicted.

#### 3.2.1. Theme (1) Planning and Time Management

Expectations: Most participants mentioned lack of time, due to the CRT schedule, appointments with health professionals, and travelling, as possible barriers to attend training sessions and to complete the exercise intervention (quote nr. 1). The interaction between time-consuming CRT treatment schedule with a patients’ daily life schedule was also mentioned as a barrier for being able to perform the exercises according to the protocol (quote nr. 2). However, one participant expected to have plenty of time because he temporarily paused his (voluntary) work during treatment.

Experiences: Post-intervention, most participants confirmed that the busy treatment schedule including travelling was perceived as an important barrier for participation in the exercise intervention (quote nr. 3) and for some it was the reason for ending their participation in the exercise trial (quote nr. 4). Admittance to the hospital, planned or unplanned, was also a barrier to perform the exercises. Planning the home-based exercises at a fixed time helped some participants to comply with the intervention.

#### 3.2.2. Theme (2) Treatment Toxicity

Expectations: Some participants were uncertain whether they would be able to perform the exercises due to expected treatment toxicity, like nausea and loss of energy (quote nr. 5). Some assumed that the CRT treatment schedule and its related toxicity would negatively affect their ability to perform the exercises and/or complete the physiotherapeutic session. The combination of treatment and participating in the exercise intervention was expected as “heavy” and was assumed to require a lot of physical strength.

Experiences: Most participants confirmed that CRT toxicity, including nausea, weight loss, loss of energy, pain, and having a feeding tube, limited their adherence and compliance to the exercise program (quote nr. 6). It was mentioned that participation in the exercise program after treatment positively contributed to emotional and physical well-being (quote nr. 7).

#### 3.2.3. Theme (3) Motivation to Exercise

Expectations: The belief that being physically active during treatment could help one to stay fit and improve health outcomes or survival was an important motivational factor for some to participate (quote nr. 8). In addition, some participants mentioned feeling better, with enjoying being active as an incentive to exercise. Some participants were self-confident and mentioned that their strong willpower would help them to adhere to and complete the exercise intervention during treatment (quote nr. 9). Others expected to improve self-esteem and mental wellbeing when participating in the exercise intervention (quote nr. 10). Some mentioned their sporty attitude as a motivating factor (quote nr. 11). On the contrary, for others their lack of a sporty attitude was a reason to participate in this intervention (quote nr. 12). A positive experience with exercising during cancer treatment of peers (quote nr. 13) and having a dog to walk with were also mentioned as motivating factors. Gaining insight into personal physical performance and strength during the intervention was said to be motivating. Some participants deemed the appointment with the physiotherapist necessary to adhere to the exercises, because of a lack of intrinsic motivation.

Experiences: In general, most factors associated with motivation to exercise mentioned at baseline were confirmed post-intervention, including the persuasion of improved health outcomes and participants’ motivation not to feel and act like a patient but to “stay in control” (quote nr. 14). One participant regarded exercising as “not being absolutely necessary for his health or survival”. Because of this conviction, exercising had a low priority for him (quote nr. 15). Some experienced a lack of discipline and loss of self-control (quote nr. 16) due to treatment toxicity and related distress. Others were able to keep motivated (quote nr. 17) because of their prior exercise behaviour, their attitude (maintaining self-control) or study commitment (quotes nr. 18). Commitment to the supervised appointments was experienced as motivating increased adherence as well as having a supportive peer or partner (quote nr. 19). Having to walk a dog was also mentioned as motivating post-intervention. One participant even borrowed a dog during treatment for that reason (quote nr. 20). Most patients had no previous experience with a cancer diagnosis or treatment and, therefore, lacked insight into the possible side-effects of CRT, and how these could affect adherence to the exercise program (quote nr. 21).

#### 3.2.4. Theme (4) Exercise Intervention

Expectations: Despite receiving in-depth information about the exercise intervention, for some participants the content and goals of the exercise program were unclear before the start of the intervention (quote nr. 22). For others, the content was sufficiently clear, in particular for some participants who had previous experiences with supervised exercising. One participant deemed it feasible to perform the exercises according to the protocol as he perceived the number of exercises as acceptable. A few participants expected the exercises to be simple to perform. Some participants expected to have sufficient stamina to adhere to the program, provided that it would be adjusted to their (changing) capacity during treatment (quote nr. 23)

Experiences: Most participants perceived the simplicity of the exercise program as a facilitator, increasing the feasibility of the exercise program. The home-based setting, and not having to go to a fitness centre, lowered the threshold for performing the exercises (quote nr. 24). The home-based setting also enabled social support for one participant, as his partner performed the exercises together with him (quote nr. 25). Yet, some pre-existing physical limitations or physical barriers due to treatment toxicity were also mentioned to negatively influence exercise adherence and compliance.

#### 3.2.5. Theme (5) Supervision by the Physiotherapist

Expectations: Participants mentioned various expectations and needs regarding supervision by the physiotherapist. Some had been treated for other conditions by a physiotherapist previously and assumed that supervision by a physiotherapist would positively affect adherence and compliance with the exercises (quote nr. 26). Participants thought that a personal approach and coaching style would help to increase adherence to the physical fitness intervention. Also, participants expected that the physiotherapist would help them perform the exercises correctly (quote nr. 27), thereby increasing the effectiveness of the exercises, and allowing adjustment of the exercises to their physical abilities (quote nr. 28).

Experiences: Participants reported that guidance by a physiotherapist was important, and it was mainly experienced as being very positive and motivating. Some preferred a more directive approach, while others preferred gentle stimulation by the physiotherapist (quote nr. 29). Clear instructions were perceived as being important for increasing compliance (quote nr. 30). Physiotherapeutic supervision helped participants to challenge themselves within their personal limits of their ability (quote nr. 31). Motivation by the physiotherapist helped to perform the exercises and facilitated increasing adherence (quote nr. 32).

#### 3.2.6. Suggestions for Improvement—Comments from Participants

One participant suggested developing exercise videos instead of the pictures we used to increase compliance with the home-based strength program (quote nr. 33). Another suggestion mentioned was to enable them to choose the training facility (for the supervised sessions) at each participants’ convenience, near home or at the hospital (quote nr. 34 and 35). Some suggested that exercising in a group with peers might increase adherence (quote nr. 36). Others preferred a program which was even more adjusted to one’s fluctuating physical capacity during treatment than our current program was (quote nr. 37). Some perceived the exercises as being too challenging, while others perceived the intensity of the exercises as being too light. Finally, it was suggested to replace the pedometer with a more user-friendly activity-tracking application (quote nr. 38). Explanatory quotes are shown in Table 3.

## 4. Discussion

This qualitative study was designed to identify factors influencing adherence, retention and compliance of patients with HNC regarding a combined supervised and home-based exercise intervention during CRT. Five themes addressing preferences, expectations, experiences and satisfaction regarding the exercise intervention were identified.

Planning and time management was the first theme identified. Participants received an intensive treatment schedule, comprising radiotherapy treatment five times a week combined with three weekly admissions for chemotherapy, and appointments with several health professionals, (unplanned) hospital admissions, and, for some, travelling time was important factors negatively affecting adherence and compliance. A lack of time has also been mentioned as a barrier to exercising by HNC patients in previous studies [20,21]. To overcome planning and time-management barriers, more flexible (re-)scheduling of supervised sessions as well as training at a location of the patients’ convenience might be beneficial.

Our findings are in line with results from other exercise studies in HNC, in which fatigue, nausea and physical weakness were also mentioned as important treatment-related barriers to attending training sessions [22,23]. Treatment toxicity, the second theme in our study, was perceived as main barrier negatively affecting adherence, retention and compliance rates which was also illustrated by our quantitative data [13]. Some adjustments to the exercise program might be helpful to overcome this problem. One option, which might increase adherence, is to start supervised training sessions before treatment and focus on home-based training with remote supervision during and shortly after CRT [23]. We suggest integrating exercise interventions within the oncological care pathway and start exercising as early as possible to achieve relevant effects of the intervention in the short period before the start of treatment. To prevent exercise-induced adverse effects, we advise following the guidelines of the National Comprehensive Cancer Network for when medical clearance and/or further medical evaluation by a medical professional is indicated [8].

The third theme in our analysis identified is the motivation to exercise. The belief that exercising helps to maintain physical fitness and improves health outcomes, including survival was perceived as a facilitator to adhere to the exercise intervention. Also, mental well-being, like self-esteem and enjoying exercising were mentioned as motivating factors. Loss of self-control due to treatment and treatment-related distress were mentioned as factors decreasing motivation to exercise. High levels of distress, anxiety and depression are common in patients with HNC [24,25]. Distress, depression, and anxiety influence physical activity and compliance to exercise [26]. For any intervention to be successful, it seems necessary to adequately address these psychological factors throughout the course of treatment [14,27]. The physiotherapist can have a pivotal role in this [28]. The beneficial effects of exercise on depression, anxiety and distress have been well established [8,29], as some interviewees said that they experienced a positive mental effect of participation in this exercise intervention. Supportive partners or peers positively influenced motivation, which was also reported in previous research [30].

Theme four describes the exercise intervention. The simplicity of the program, the commitment to the supervised appointments and gaining insight into personal performance increased motivation and adherence. Factors that negatively influenced adherence were unclear expectations regarding the content and lack of goalsetting of the exercise intervention. In patients with HNC, adherence, retention and compliance to exercise interventions can be challenging because they typically have a less active lifestyle compared with other populations with cancer and have a high symptom burden [23,31]. The exercise intervention was tailored to patients’ capacity and preference for endurance training throughout the program. However, physical limitations and perceived insufficient adjustment of the intensity of the program were experienced as barriers. Consequently, adherence, retention and compliance may be increased with more extensive adjustment of the exercise intervention based on physical limitations, and by setting personal goals [32]. More time is, therefore, needed for supervision and guidance by a physiotherapist during the exercise program. The home-based part of our exercise program was mentioned as a facilitator for high compliance. In addition, group training sessions might increase motivation in patients with HNC, as has been shown in a previous study [22]; although, this is difficult to achieve in a peripheral setting due to the low prevalence of HNC in The Netherlands.

The last and fifth theme identified was supervision by a physiotherapist. Supervision by a physiotherapist was deemed necessary for proper instructions in performing the exercises correctly and increasing motivation and compliance. As shown in previous studies, the physiotherapist has an important facilitating role in motivation, mental support and increasing discipline to exercise and supervised exercise programs [33,34].

The scope of this study was to only include patients who participated in the exercise intervention, and only one participant had a low education level, which is not representative of the entire HNC population. We assume that the presence of the partner of two interviewees during their interviews will not have affected the reliability of our results. In our opinion, it might positively affect validation as the interviewee felt more at ease and thus provided more extensive responds. Participants of our study are likely to be more active and might have different beliefs and preferences to non-participants, resulting in selection bias, as has been previously described [13,35].

### Clinical Implications and Future Directions

The current exercise program was adapted to the participants’ capacity; however, some expected a more tailored intervention. An optimal personalized intervention with regard to goal-setting, training type, intensity, setting, and timing might further increase feasibility outcomes. A previous study showed that, to increase physical activity levels in HNC, exercise should preferably be incorporated in daily life activities [9]. As the normal structure of daily life activities is changed due to the intensive treatment schedule, future studies should focus on how to flexibly (re-)schedule the supervised training sessions. Exercise programs should preferably be offered as part of usual care with training sessions scheduled around treatment appointments. This would overcome some of the logistic barriers, as well as the low adherence due to treatment toxicity. In a previous qualitative study in HNC survivors, a lack of intention to increase their physical activity level was reported, due to the incorrect assumption that their current physical activity level was already sufficient [9]. The assumption of “already being active” was also an important reason for not willing to participate in this exercise intervention [13]. E-health applications or blended care can be helpful in providing patient-tailored information on activity level, personal goals and monitoring of individual progress [36,37], as was also suggested by the interviewees.

## 5. Conclusions

In conclusion, five themes, planning and time management, treatment toxicity, motivation to exercise, exercise intervention and supervision by the physiotherapist, were identified. A personalised approach, considering the individual facilitators and barriers within these themes, is important to increase the feasibility of exercise intervention during HNC treatment and to reach optimal physical fitness effects.

## Figures and Tables

**Table 1:** Baseline characteristics of the 14 participants.

Participant No.	Age (years)	Gender	Educational Level ^1^	Disease Stage	Tumor Location	Number of Attended Supervised Sessions (out of 10)	Completed the Intervention	Interview before Intervention	Interview after Intervention	Rating Exercise Intervention ^2^
1	58	M	Middle	IV	Oro- pharynx	4	yes	yes	yes	8
2	56	M	High	III	Oro- pharynx	2	no	yes	yes	missing
3	35	M	Middle	IV	Oral cavity	9	yes	yes	yes	8
4	65	F	Middle	IV	Oro- pharynx	2	no	yes	yes	8
5	60	M	Middle	V	Oro- pharynx	9	yes	yes	yes	6
6	47	M	Middle	III	Oro- pharynx	2	no	yes	yes	8
7	63	M	Middle	IV	Oral cavity	7	yes	yes	yes	8
8	70	M	Low	IV	Hypopharynx	3	no	yes	no	missing
9	59	F	High	IV	Oro- pharynx	10	yes	yes	yes	8
10	58	M	Middle	III	Oro- pharynx	8	yes	yes	yes	5
11	53	F	High	III	Oro- pharynx	4	no	yes	yes	7.5
12	67	M	High	IV	Oral cavity	8	yes	yes	yes	10
13	54	M	Middle	IV	Oral cavity	4	yes	yes	no	missing
14	56	M	High	IV	Oro- pharynx	10	yes	yes	yes	7.5

^1^ Low, primary school or lower general secondary or prevocational secondary education; Middle, upper secondary education or vocational training; High, higher education or university. ^2^ On a scale between 0 and 10 participants were asked to rate satisfaction with the exercise intervention.

**Table 2:** Quotes illustrating patients’ expectations and experiences of exercise intervention per theme and subthemes.

Themes	Subthemes	Quotes (Patient nr, Quote nr)
**Planning and time** **management**	**Expectations**	
	Lack of time due to chemoradiotherapy schedule	“I hesitated because I already saw my agenda filling up completely with all sorts of different things.” (patient nr. 12, quote nr. 1)
	Structure of daily life activities	“My daily routine is completely thrown off, you are not in charge of your own calendar anymore, so it is a bit of a puzzle where to fit this in, but then again this number of exercises should not make this impossible.” (patient nr 2, quote nr. 2)
	**Experiences**	
	Intensity of chemoradiotherapy schedule	“You do not know what hits you. You must see the dental hygienist, the dietitian, the speech therapist… In the month of May, we had over 50 appointments scheduled at the hospital.” (patient nr. 4, quote nr. 3)
	Intensity of chemoradiotherapy schedule, travelling time	“And the reason for dropping out, that had to do with, like, there is so much you have to deal with when starting [therapy], you hardly realize what you agreed to. The intensity of the program and all that comes with it, not just the program, but having to travel more than three hours every day to get to and from the hospital. And then also having to comply to this program, in combination with all kinds of other appointments, that made it too hard.” (patient nr. 2, quote nr. 4)
**Treatment** **toxicity**	**Expectations**	
	Intensity of chemoradiotherapy treatment, physical ability	“I can imagine, that when you have just had your chemotherapy treatment, and you are extremely nauseated. Well, then, of course, it becomes difficult to motivate yourself and actually perform the exercises.” (patient nr. 10, quote nr. 5)
	**Experiences**	
	Nausea, weight loss, energy loss, pain, tube feeding	“At one point I could not stop throwing up… in a few days I became scrawny. It terrified me. Then I was admitted to the hospital. So, then you’re not like; okay, I should go ahead and do my exercises now.” (patient nr. 11, quote nr. 6)
	Emotional well-being	“You are happy after that last radiotherapy treatment; it’s over, you could just kiss everyone. But then you fall into a void, and then it is nice that there still is this exercise program, with its weekly appointments with the physiotherapist, so there was at least that, so this was especially helpful mentally. (patient nr. 4, quote nr. 7)
**Motivation** **to exercise**	**Expectations**	
	Physical health benefits	“Motivation to survive, and also a shorter rehabilitation period, but initially, strive to survive. So, everything I can do to support this treatment I will do. ” (patient nr. 3, quote nr. 8)
	Willpower	“There is no such thing as “I can’t do this anymore”, never ever, I can always take it a step further, at least at my level you can always take it a step further. The average top athlete will not be able to run much faster, but in my condition, there is always room for improvement” (patient nr. 3, quote nr. 9)
	Confidence	“Self-esteem, increasing confidence. I guess. Feeling less of a pitiful little creature… feeling better both physically and mentally being more confident. “ (patient nr 12, quote nr. 10)
	Sporty attitude	“Anyway, I already had the intention [to exercise] in advance. If you exercise on a regular basis during treatment, that’s just better. You pull through easier, you are fitter, you might have less drug side-effects and so on.” (patient nr. 10, quote nr. 11)
	Lack of sporty attitude	“Well, actually, I must confess I am a bit, ehm, this is anonymous right? I am actually very lazy.” (patient nr. 9, quote nr. 12)
	Peers, experiences of peers	“The experiences of someone I know, who has also had cancer, breast cancer, she told me she stayed as active as she could and this helped her a lot. And she is about my age, a few years younger, so I thought: that is a valuable piece of advice. And that’s how I selected tips and advice from people around me every now and then.” (patient nr. 9, quote nr. 13)
	**Experiences**	
	Physical health benefits	“I am convinced that, ehm, that for my recovery and maybe also to prevent deterioration, exercising is simply very beneficial. That is sort of what I think.” (patient nr. 10, quote nr. 14)
	Health beliefs, attitude	“You are also just tired of being ill, so the things that are not absolutely necessary for your health or to survive… well… they can wait until tomorrow.” (patient nr. 5, quote nr. 15)
	Loss of self-control	“at one point I was extremely nauseated, I just did not perform the exercises anymore, I just couldn’t. But I did take it up again, one week later. But it did give me a bit of a scare, because I often don’t know my own boundaries, so I became scared and then I dropped out”. (patient nr. 12, quote nr. 16)
	Self-control	“And everything I can do to feel less like a patient and to speed up my recovery I will do! So I was quite motivated not to be discouraged and not to become a passive patient, but instead keeping self-control during the treatment trajectory as well as during the rehabilitation phase.” (patient nr. 14, quote nr. 17)
	Physical health benefits	“Sitting is the new smoking”, they say, and not without reason, so considering that, and especially in these extreme circumstances, it is just good to do it [exercising].” (patient nr. 9, quote nr. 18)
	Commitment to the study program	“I already intended to exercise, even if I would not have participated in this study, as I had said before. Anyway, I still would have planned to do something, so that was my motivation. And then it is just discipline, especially when you are not feeling well.” (patient nr. 10, quote nr. 19)
	Chemo dog, social support	“I deliberately borrowed a dog during my treatment, to arrange my physical activity routine.” (patient nr. 14, quote nr. 20)
		“Fortunately, I have little experience with cancer. This is the first time, but you just have no clue… There is so much coming at you, it is very difficult to predict whether it will be feasible [exercising]”. (patient nr. 2, quote nr. 21)
**Exercise** **intervention**	**Expectations**	
	Content, unclear expectations	“… I don’t know what to expect, so maybe… I don’t know what we are going to do yet.” (patient nr. 6, quote nr. 22)
	Personalized intervention	“Yes well, I would assume that, when developing the program, you gave it some consideration that one should be able to keep it up”. (patient nr. 9, quote nr. 23)
	**Experiences**	
	Simplicity of the intervention, home-based	“The simplicity, that is of course the strength of this program, anyone can do it, you don’t have to go to the gym. You can just do it at home whenever you want. It is simple, and that, of course, is the strength of this program. Because, if you’re aiming for feasibility you should not add the constraint that one must go to the gym.” (patient nr. 10, quote nr. 24)
	Home-based, social support	“It took me a while to get into it (home-based exercises), because I’m not used to that, but later on I did it together with my wife. She also got one of those (resistance) bands, and then we did it together, she is really good at it”. (patient nr. 12, quote nr. 25)
**Supervision** **physiotherapist**	**Expectations**	
	Coaching, motivating	“Well a strong external incentive, I definitely need that, because I think I am rather lazy by nature.” (patient nr. 9, quote nr. 26)
	Coaching, performance	“By correcting me when I didn’t perform the exercises properly. You know, of course I did them once and I have seen those pictures, but the correct posture… that is hard. You tend to make it as easy on yourself as possible with those exercises, but you have to adopt the right posture that truly makes you put in the effort.” (patient nr. 5, quote nr. 27)
	Coaching, personal approach	“In any case, it offers me (ehm) a custom-fit solution to stay sportive, or at least physically active”. (patient nr. 14, quote nr. 28)
	**Experiences**	
	Personal approach, coaching	“That physiotherapist, yeah, I think she put too much pressure on me… to go, (ehm)… to the extreme… for me that works counterproductive”. (patient nr. 11, quote nr. 29)
	Clear instructions	“You can do the exercises in many different ways, and there was actually only one good way. The physiotherapist was always very pleased that I remembered the exercises well and performed them in the correct way.”(patient nr. 5, quote nr. 30)
	Personal approach, coaching	“Yes, I found it very stimulating, really empowering, (ehm) the physiotherapist was really driven, and you become aware of what your limits are, and what you can still do…”. (patient nr. 12, quote nr. 31)
		“I think it is truly fantastic! Very well done, inspired, and the physiotherapist is of course a wonderful person, but also the way she presented it and made it attractive by stimulating me, yeah, that is really the way to get someone moving”. (patient nr. 12, quote nr. 32)

**Table 3:** Quotes illustrating patients’ suggestions for improvement of the exercise intervention.

Suggestions for Improvement	Exemplary Quotes
Exercise videos	“What might be helpful,… you know, I had to do 6 different exercises… and if there were like 6 YouTube videos with exactly those exercises”. (patient nr. 5, quote nr. 33)
Supervised training near home	“I think, yeah, if a physiotherapist had visited me at home, I probably would have done those exercises”. (patient nr. 6, quote nr. 34)
Hospital-based training	“During hospital stay, I really liked it, but at home there was so much going on, too many distractions, and all the hassle with medication, tube feeding, that made it impossible to also do it (the exercises) on top of all that”. (patient nr. 5, quote nr. 35)
Exercise in peer group	“I think it is better (to exercise) in a group”. (patient nr. 4, quote nr. 36)
Personalized training program	“Consider each individuals’ own personal needs. I had a need for a more intensive program and with that, I would have liked the freedom to adjust the exercises when it’s not going well on occasion”. (patient nr. 10, quote nr. 37)
Health/exercise tracking apps	“To be honest, I didn’t find the fitbit very convenient,… I think it would be better to use your smartphone for tracking, because you always have it on you,… you know. I change my trousers before leaving the house and then the fitbit was still attached to the house pair…”. (patient nr. 5, quote nr. 38)

## Data Availability

The data that support the findings of this study are available from the corresponding author upon reasonable request.

## References

[1] Ameri A., Norouzi S., Sourati A., Azghandi S., Novin K., Taghizadeh-Hesary F. (2022). Randomized trial on acute toxicities of weekly vs. three-weekly cisplatin-based chemoradiation in head and neck cancer. Cancer Rep..

[2] Szturz P., Wouters K., Kiyota N., Tahara M., Prabhash K., Noronha V., Castro A., Licitra L., Adelstein D., Vermorken J.B. (2017). Weekly Low-Dose Versus Three-Weekly High-Dose Cisplatin for Concurrent Chemoradiation in Locoregionally Advanced Non-Nasopharyngeal Head and Neck Cancer: A Systematic Review and Meta-Analysis of Aggregate Data. Oncologist.

[3] Van den Bosch L., van der Laan H.P., van der Schaaf A., Oosting S.F., Halmos G.B., Witjes M.J.H., Oldehinkel E., Meijer T.W.H., van den Hoek J.G.M., Steenbakkers R.J.H.M. (2021). Patient-Reported Toxicity and Quality-of-Life Profiles in Patients With Head and Neck Cancer Treated with Definitive Radiation Therapy or Chemoradiation. Int. J. Radiat. Oncol. Biol. Phys..

[4] Silver H.J., Dietrich M.S., Murphy B.A. (2007). Changes in body mass, energy balance, physical function, and inflammatory state in patients with locally advanced head and neck cancer treated with concurrent chemoradiation after low-dose induction chemotherapy. Head. Neck..

[5] Rogers L.Q., Courneya K.S., Robbins K.T., Malone J., Seiz A., Koch L., Rao K., Nagarkar M. (2006). Physical activity and quality of life in head and neck cancer survivors. Support. Care Cancer.

[6] Ligibel J.A., Bohlke K., May A.M., Clinton S.K., Demark-Wahnefried W., Gilchrist S.C., Irwin M.L., Late M., Mansfield S., Marshall T.F. (2022). Exercise, Diet, and Weight Management During Cancer Treatment: ASCO Guideline. J. Clin. Oncol..

[7] Patel A.V., Friedenreich C.M., Moore S.C., Hayes S.C., Silver J.K., Campbell K.L., Winters-Stone K., Gerber L.H., George S.M., Fulton J.E. (2019). American College of Sports Medicine roundtable report on physical activity, sedentary behavior, and cancer prevention and control. Med. Sci. Sports Exerc..

[8] Campbell K.L., Winters-Stone K.M., Wiskemann J., May A.M., Schwartz A.L., Courneya K.S., Zucker D., Matthews C., Ligibel J., Gerber L. (2019). Exercise Guidelines for Cancer Survivors: Consensus Statement from International Multidisciplinary Roundtable. Med. Sci. Sports Exerc..

[9] Sealy M.J., Stuiver M.M., Midtgaard J., van der Schans C.P., Roodenburg J.L.N., Jager-Wittenaar H. (2021). Perception and Performance of Physical Activity Behavior after Head and Neck Cancer Treatment: Exploration and Integration of Qualitative and Quantitative Findings. Int. J. Environ. Res. Public Health.

[10] D’Souza M., Samuel S.R., Saxena P.P. (2020). Effects of Exercise Training during Concomitant Chemoradiation Therapy in Head-and-Neck Cancer Patients: A Systematic Review. Indian J. Palliat. Care..

[11] Lonkvist C.K., Lonbro S., Vinther A., Zerahn B., Rosenbom E., Primdahl H., Hojman P., Gehl J. (2017). Progressive resistance training in head and neck cancer patients during concomitant chemoradiotherapy—Design of the DAHANCA 31 randomized trial. BMC Cancer.

[12] Samuel S.R., Maiya A.G., Fernandes D.J., Guddattu V., Saxena P.U.P., Kurian J.R., Lin P.-J., Mustian K.M. (2019). Effectiveness of exercise-based rehabilitation on functional capacity and quality of life in head and neck cancer patients receiving chemo-radiotherapy. Support. Care Cancer.

[13] Kok A., Passchier E., May A.M., van den Brekel M.W., Jager-Wittenaar H., Veenhof C., de Bree R., Stuiver M.M., Speksnijder C.M. (2022). Feasibility of a supervised and home-based tailored exercise intervention in head and neck cancer patients during chemoradiotherapy. Eur. J. Cancer Care.

[14] Ning Y., Wang Q., Ding Y., Zhao W., Jia Z., Wang B. (2022). Barriers and facilitators to physical activity participation in patients with head and neck cancer: A scoping review. Support. Care Cancer.

[15] Buffart L.M., de Bree R., Altena M., van der Werff S., Drossaert C.H.C., Speksnijder C.M., van Den Brekel M.W., Jager-Wittenaar H., Aaronson N.K., Stuiver M.M. (2018). Demographic, clinical, lifestyle-related, and social-cognitive correlates of physical activity in head and neck cancer survivors. Support. Care Cancer.

[16] Tong A., Sainsbury P., Craig J. (2007). Consolidated criteria for reporting qualitative research (COREQ): A 32-item checklist for interviews and focus groups. Int. J. Qual. Health Care.

[17] Creswell J.W., Poth C.N. (2017). Qualitative Inquiry and Research Design: Choosing among Five Approaches.

[18] Guest G., Bunce A., Johnson L. (2006). How Many Interviews Are Enough?:An Experiment with Data Saturation and Variability. Field Methods.

[19] Braun V., Clarke V. (2006). Using thematic analysis in psychology. Qual. Res. Psychol..

[20] Sandmael J.A., Bye A., Solheim T.S., Stene G.B., Thorsen L., Kaasa S., Lund J., Oldervoll L.M. (2017). Feasibility and preliminary effects of resistance training and nutritional supplements during versus after radiotherapy in patients with head and neck cancer: A pilot randomized trial. Cancer.

[21] Felser S., Behrens M., Liese J., Strueder D.F., Rhode K., Junghanss C., Grosse-Thie C. (2020). Feasibility and Effects of a Supervised Exercise Program Suitable for Independent Training at Home on Physical Function and Quality of Life in Head and Neck Cancer Patients: A Pilot Study. Integr. Cancer Ther..

[22] Jackson C., Dowd A.J., Capozzi L.C., Bridel W., Lau H.Y., Culos-Reed S.N. (2018). A turning point: Head and neck cancer patients’ exercise preferences and barriers before and after participation in an exercise intervention. Eur. J. Cancer Care.

[23] Capozzi L.C., McNeely M.L., Lau H.Y., Reimer R.A., Giese-Davis J., Fung T.S., Culos-Reed S.N. (2016). Patient-reported outcomes, body composition, and nutrition status in patients with head and neck cancer: Results from an exploratory randomized controlled exercise trial. Cancer.

[24] Hammermuller C., Hinz A., Dietz A., Wichmann G., Pirlich M., Berger T., Zimmermann K., Neumuth T., Mehnert-Theuerkauf A., Wiegand S. (2021). Depression, anxiety, fatigue, and quality of life in a large sample of patients suffering from head and neck cancer in comparison with the general population. BMC Cancer.

[25] Williams C. (2017). Psychosocial Distress and Distress Screening in Multidisciplinary Head and Neck Cancer Treatment. Otolaryngol. Clin. N. Am..

[26] Kampshoff C.S., van Mechelen W., Schep G., Nijziel M.R., Witlox L., Bosman L., Chinapaw M.J.M., Brug J., Buffart L.M. (2016). Participation in and adherence to physical exercise after completion of primary cancer treatment. Int. J. Behav. Nutr. Phys. Act..

[27] Ghazali N., Roe B., Lowe D., Tandon S., Jones T., Brown J., Shaw R., Risk J., Rogers S.N. (2017). Screening for distress using the distress thermometer and the University of Washington Quality of Life in post-treatment head and neck cancer survivors. Eur. Arch. Otorhinolaryngol..

[28] Wijma A.J., Bletterman A.N., Clark J.R., Vervoort S., Beetsma A., Keizer D., Nijs J., Van Wilgen C.P. (2017). Patient-centeredness in physiotherapy: What does it entail? A systematic review of qualitative studies. Physiother. Theory Pract..

[29] Singh B., Olds T., Curtis R., Dumuid D., Virgara R., Watson A., Szeto K., O’Connor E., Ferguson T., Eglitis E. (2023). Effectiveness of physical activity interventions for improving depression, anxiety and distress: An overview of systematic reviews. Br. J. Sports Med..

[30] McDonough M.H., Beselt L.J., Kronlund L.J., Albinati N.K., Daun J.T., Trudeau M.S., Wong J.B., Culos-Reed S.N., Bridel W. (2021). Social support and physical activity for cancer survivors: A qualitative review and meta-study. J. Cancer Surviv..

[31] Wells M., King E. (2017). Patient adherence to swallowing exercises in head and neck cancer. Curr. Opin. Otolaryngol. Head. Neck Surg..

[32] Rogers S.N., Lowe D., Midgley A.W. (2022). Patients’ views of physical activity whilst living with and beyond head and neck cancer. Int. J. Oral. Maxillofac. Surg..

[33] van Vulpen J.K., Witlox L., Methorst-de Haan A.C., Hiensch A.E., van Hillegersberg R., Ruurda J.P., Nieuwenhuijzen G.A.P., Kouwenhoven E.A., Siersema P.D., May A.M. (2023). Perceived facilitators and barriers by esophageal cancer survivors participating in a post-treatment exercise program. Support. Care Cancer.

[34] Buffart L.M., Kalter J., Sweegers M.G., Courneya K.S., Newton R.U., Aaronson N.K., Jacobsen P.B., May A.M., Galvao D.A., Chinapaw M.J. (2017). Effects and moderators of exercise on quality of life and physical function in patients with cancer: An individual patient data meta-analysis of 34 RCTs. Cancer Treat. Rev..

[35] Reynolds S.A., O’Connor L., McGee A., Kilcoyne A.Q., Connolly A., Mockler D., Guinan E., O’Neill L. (2023). Recruitment rates and strategies in exercise trials in cancer survivorship: A systematic review. J. Cancer Surviv..

[36] van der Meer H.A., de Pijper L., van Bruxvoort T., Visscher C.M., Nijhuis-van der Sanden M.W.G., Engelbert R.H.H., Speksnijder C.M. (2022). Using e-Health in the physical therapeutic care process for patients with temporomandibular disorders: A qualitative study on the perspective of physical therapists and patients. Disabil. Rehabil..

[37] Toonders S.A.J., van der Meer H.A., van Bruxvoort T., Veenhof C., Speksnijder C.M. (2022). Effectiveness of remote physiotherapeutic e-Health interventions on pain in patients with musculoskeletal disorders: A systematic review. Disabil. Rehabil..

